# Prospective assessment of subjective sleep benefit in Parkinson’s disease

**DOI:** 10.1186/s12883-014-0256-2

**Published:** 2015-01-16

**Authors:** Merel M van Gilst, Bastiaan R Bloem, Sebastiaan Overeem

**Affiliations:** Department of Neurology, Radboud University Medical Centre, Donders Institute for Brain, Cognition and Behaviour, Nijmegen, The Netherlands; Sleep Medicine Centre Kempenhaeghe, Heeze, The Netherlands

**Keywords:** Sleep, Parkinson’s disease, Sleep benefit, Nap, Questionnaire

## Abstract

**Background:**

Parkinson’s disease (PD) patients may experience ‘sleep benefit’ (SB): a temporarily improved mobility upon awakening. SB has mainly been studied retrospectively using questionnaires, but it remains unclear whether it is associated with actual changes in motor functioning.

**Methods:**

We performed a prospective study on sleep-related changes in motor functioning, using a PD symptom diary during 7 days in 240 randomly selected PD patients (140 men; 66.8 ± 9.6 years; disease duration 9.3 ± 6.2 years). Afterwards, patients received a questionnaire on the possible subjective experience of SB.

**Results:**

Using the PD symptom diary, a positive change in motor function was observed after 267 nights (17.8%) and after 138 daytime naps (23.4%). Based on these results, 75 patients (32%) were classified as having SB. In response to the subsequent questionnaire, 73 patients (31%) reported SB. Interestingly, the groups with SB according to either the diary or the questionnaire overlapped only partially: outcomes were congruent in 63% of subjects (both negative 49%, both positive 14%). In both the diary and questionnaire, patients with SB showed a longer disease duration and longer medication use. According to the questionnaire, there was a trend towards a shorter sleep duration and lower sleep efficiency in the SB group. The mean change in motor function after sleep as assessed using the diary was higher in patients reporting subjective SB.

**Conclusion:**

We show that the subjective experience of SB in PD is not always related to an actual increase in reported motor function after sleep. Defining SB using either a symptom diary or a questionnaire on subjective experience, results in only partly overlapping groups. These data suggest that SB may be a more heterogeneous phenomenon than previously thought and that subjective experience of symptom severity is not necessarily related to actual motor function.

**Electronic supplementary material:**

The online version of this article (doi:10.1186/s12883-014-0256-2) contains supplementary material, which is available to authorized users.

## Background

Sleep in Parkinson’s disease (PD) is generally associated with sleep *disorders*, which affect 60-98% of PD patients [1-3]. However, clinicians have repeatedly noted reports of patients experiencing a Parkinson-specific *beneficial* effect of sleep. Contrary to what would be expected after a night without medication, some patients describe a temporarily improved mobility upon awakening. In the early eighties, Marsden was the first to describe this phenomenon, coining the term “sleep benefit” (SB) [4]. Subjective patient reports suggest that the effect of SB may be rather pronounced, with patients sometimes comparing SB to the “on” state as induced by dopaminergic medication. Some PD patients can even delay or skip their morning dose of medication because of SB [5]. Although typically described after nocturnal sleep, SB may also occur after daytime naps [5-8]. In this study, we assess SB using a recently suggested, explicit definition: *SB is the experience of a temporary decrease in PD symptoms upon awakening after a period of sleep (night or daytime), before drug intake; the patient is feeling as good as “on” (or better)* [9]*.*

In most studies, questionnaires have been used to evaluate the prevalence and characteristics of SB. Results showed a rather consistent prevalence estimate, ranging from 33 to 55% of PD patients who recognized the phenomenon [9]. However, it remains unknown why some PD patients experience SB while others do not. Possible determinants of SB were assessed by several research groups, including age, age at onset, disease duration and presence of motor fluctuations [5,6,8,10-12]. Results varied widely and no consistent SB-associated variables have been found to date. One explanation for this may be the prominent differences in the definition of SB that were applied in the various studies [9]. Furthermore, patients often have difficulties giving an accurate account of their general motor functioning after sleep [13].

Here we performed a prospective study in a large group of PD patients using tailored symptom diaries. In the diary, patients provided an indication of specific motor functioning before and directly after every period of sleep. Using this approach, we obtained sleep-related changes in subjective motor functioning over multiple days, without explicitly asking patients about the phenomenon of SB. The objective of this study was to gain more insight into the nature of SB and the magnitude of its effect.

## Methods

### Subjects

Information letters about the study were sent to 475 PD patients under treatment at the Parkinson Centre Nijmegen and the associated national ParkinsonNet. Subsequently, these patients were contacted by telephone to provide general study information and - when interested - to book an appointment for completing the SB-diary. After telephone consultation, 292 patients agreed to participate, of which 240 patients completed both the diary and questionnaire. The study was approved by the Medical Ethical Committee of the Radboud university medical centre. All patients provided written informed consent to participate.

### Diary

The symptom diary was based on the SCOPA Diary Card (SCOPA DC) [14], a validated instrument to assess changes in motor functioning during the day. Before and directly after every night of sleep and every daytime nap, patients answered four questions with respect to PD-related motor functioning. The diary was completed at bedtime and directly at awakening, before medication intake. Patients indicated on a four-point scale how well they could perform three activities, i.e. walking, changing position and using their hands. These activities have proven to give a reliable indication of general motor functioning [14]. In addition, the question on sleep quality from the SCOPA DC was used and we added an extra question on feeling rested upon awakening, all recorded using four-point Likert scales.

Medication taken within 2 hours before a period of sleep or during the night-time was recorded in the diary. All dopaminergic medication was converted into the L-dopa equivalent dose (LED), using the formula described by Tomlinson et al. [15].

Patients completed the symptom diary during one week, before and directly after every period of sleep (both night sleep and daytime naps). Importantly, in this phase of the study, patients were not explicitly informed about the phenomenon of SB.

### Questionnaire

After completing the diary week, patients received an extensive questionnaire, in which demographic characteristics and clinical information were obtained. The Pittsburgh Sleep Quality Index (PSQI) provided a validated measure of nocturnal sleep quality during the past month [16], therefore the questionnaire had to be completed within a month after the symptom diary.

Subsequently, a brief explanation of SB was given in the questionnaire. This explanation of SB was based on the revised definition of SB recently presented by our group [9]. Afterwards, patients were asked whether they were familiar with SB, and if yes, how often they experienced SB. Finally, information was obtained on the duration, degree of improvement and variability of the SB experience (see Additional file 1 for the translated SB questionnaire).

### Analyses

For all analyses, night sleep and daytime naps were regarded separately. Motor functioning was defined as the sum of the three functioning questions in the diary (i.e. walking, changing position, using hands). Changes in motor function in relation to sleep were calculated by subtracting post-sleep motor scores from pre-sleep motor functioning. This was done for every period of sleep separately.

A night with a positive change (better functioning in the morning than in the evening) was regarded as a “SB-night”. The same approach was used for daytime naps. Patients were classified as having SB, when SB was present after at least 2 nights or 2 naps during the 7 day period.

All analyses were performed in SPSS for Windows version 20. Groups with and without SB were compared using t-tests or chi^2^-tests. Correlations were analyzed using Spearman’s correlations. Alpha was set to 0.01 to correct for multiple comparisons.

## Results

Of the 292 participating patients, a total of 240 completed the 7-day motor diary and the subsequent SB questionnaire. Mean age was 66.8 ± 9.6 years (range 33–94 years) and the number of men was 140 (58.3%). Mean disease duration was 9.3 ± 6.2 years (range 1–35 years), with a mean age of PD onset of 57.4 ± 12.2 years (range 22–87 years). The most important reasons for dropout were: illness (25), patients considering the symptom diary too confronting and/or demanding (16) and being lost to follow up (9). Patients dropping out were older than those who finished the diary (72.3 ± 10.6 vs. 66.8 ± 9.6 years, p < 0.001) but there were no gender differences between the groups.

### SB diary

In 5 diaries more than 50% of the questions were missing; these diaries were excluded from the analyses. Valid information was obtained from a total of 1496 nights (91%) and 591 (90%) naps (mean duration 21.7 ± 45.9 min). A positive change in motor function was observed after 267 nights (17.8%), and after 138 naps (23.4%). Based on these results, 76 patients (32%) were classified as having SB, 44 patients (58%) only showed sleep benefit after night sleep, 11 (14%) only after an afternoon nap and 21 patients (28%) after both. None of the patients had SB after every night of the week. In Table 1, the number of patients are listed with the number of nights associated with a positive change in motor function, over the measurement week. When comparing patients with and without SB based on the symptom diary, we found that patients with SB had used PD medication during a longer period of time and showed a trend towards longer disease duration (Table 2). There were no differences in gender distribution, age, or sleep quality between patients with and without SB.

**Table 1:** **Patient report of total number of nights per week with SB according to the PD symptom diary**

**Number of SB nights per week**	**Patients n(%)**
**0**	111 (47.2%)
**1**	59 (25.1%)
**2**	25 (10.6%)
**3**	19 (8.1%)
**4**	8 (3.4%)
**5**	9 (3.8%)
**6**	4 (1.7%)
**7**	0 (0%)

**Table 2:** **Characteristics of patients with and without SB**

	**SB based on diary**			**SB based on questionnaire**		
	**SB**	**no-SB**	**p**	**SB**	**no-SB**	**p**
**SB**	76 (32%)	159 (68%)		74 (31%)	163 (69%)	
**Men**	43 (57%)	94 (59%)	0.741	37 (50%)	102 (63%)	0.068
**Age** (yrs)	67.3 ± 9.7	66.3 ± 9.6	0.454	63.6 ± 8.2	68.5 ± 9.7	0.000*
**Age PD onset** (yrs)	56.6 ± 13.6	57.5 ± 11.5	0.587	51.9 ± 11.1	60.0 ± 11.9	0.000*
**Duration of PD symptoms** (yrs)	10.8 ± 7.0	8.7 ± 5.8	0.019	11.7 ± 6.7	8.2 ± 5.7	0.000*
**Duration medication use** (yrs)	9.0 ± 6.0	6.8 ± 5.5	0.009*	9.4 ± 6.3	6.5 ± 5.3	0.001*
**Daily LED** (mg)	752 ± 608	624 ± 409	0.080	804 ± 614	584 ± 382	0.002*
**PSQI**	7.5 ± 3.9	7.2 ± 3.9	0.680	7.8 ± 4.1	7.0 ± 3.8	0.178
**Sleep duration** (hrs - PSQI)	6.7 ± 1.3	6.7 ± 1.6	0.960	6.4 ± 1.3	6.8 ± 1.5	0.039
**Sleep efficiency** (% - PSQI)	78.7 ± 16.1	78.2 ± 17.7	0.840	74.9 ± 14.7	80.3 ± 17.9	0.027

Diary – 5 missing, Questionnaire – 3 missing, *significant difference at α = 0.01.

In the SB group, 22 patients (28.9%) used long-acting L-dopa and 5 patients (6.6%) used dopamine agonists in the 2 hours before bedtime. In the no-SB group, 30 patients (19.1%) used long-acting L-dopa and 13 patients (8.3%) used dopamine agonists in the 2 hours before bedtime. Benzodiazepines were sometimes used by 9 SB patients (12%) and 18 no-SB patients (11.5%) and other sleep modulating drugs were used by 11 patients with SB (14.7%) and 18 patient without SB (11.5%). For none of these drugs there was a significant between-group difference.

When looking at individual nights, we found no significant correlations between the amount of overnight change in motor functioning and self-rated sleep time, sleep quality or the feeling of being rested upon awakening. In addition, we found no influence of the amount of dopaminergic medication taken before and/or during the night on the occurrence of SB. For the daytime naps, no association was found between the amount of functioning change after sleep and sleep time, sleep quality, restorative feeling or dopaminergic medication taken within 2 hours before the nap.

### SB questionnaire

In the questionnaire, 74 patients (31%) indicated to experience SB. When patients were categorized based on their own subjective judgment on the presence of SB in the questionnaire, we found that the SB group not only had a longer disease duration and a longer history of medication use, but also a higher daily L-dopa equivalent dose (Table 1). In addition, patients reporting SB were younger, and had an earlier age of onset of PD. There were no differences in overall subjective sleep quality as assessed by the PSQI between the SB and no-SB group, although there was a trend towards a shorter sleep duration as well as a lower sleep efficiency in patients reporting SB (Table 1).

Additional information on the characteristics of subjective SB were obtained in the questionnaire as well. Of the patients recognizing SB, the majority reported to regularly experience SB after sleep. Strikingly, 28% of patients even reported that they always experienced SB. The mean estimated duration of SB was 1:03 ± 0:53 hours. At awakening, 47.1% reported to feel “as good as on medication” and 22.9% reported to feel even better. The remaining 30% of patients experienced an improvement in functioning, but perceived it not as good as when on medication. About a third of the subjects (31.5%) indicated to experience SB *during* the night, for example when going to the toilet. Many patients had the feeling that sleeping longer (47.3%) or shorter (58.1%) had an influence on the occurrence of SB.

We also asked patients experiencing SB to give their own written description of how they feel at waking up. Some patients gave very characteristic and specific descriptions fitting with SB. However, there were also patients who rated themselves as having SB, who provided a much more general description of feeling improved at awakening, which would fit more with a-specific refreshing effects of sleep (see “Patients description of feeling at awakening when experiencing sleep benefit”).

### Patients description of feeling at awakening when experiencing sleep benefit

#### Descriptions characteristic for SB

- *“At awakening I feel less rigid, I feel I could skip my medication if I want to”*
- *“After a nap I move faster, more flexible and more natural”*
- *“After sleeping I feel like I don’t have PD, even my handwriting is as good as it used to be”*
- *“Sometimes I forget to take my morning medication, because I feel so good”*
- *“For me sleep is the best drug against PD”*
- *“After a nap it feels like my body is reset”*
- *“At awakening in the morning I can go to the toilet by myself, whereas during the rest of the day I need help with everything”*

#### Descriptions non-characteristic for SB (i.e. general a-specific sleep effects)

- *“I feel very rested, however, I’m still very rigid”*
- *“I feel less tired after an afternoon nap”*
- *“In the morning I have more energy than in the afternoon”*
- *“After sleeping I feel more relaxed”*

### Comparing SB diary and questionnaire outcomes

Interestingly, the group of patients classified as having SB based on the symptom diary, overlapped only partially with the group of patients self-reporting to have SB in the questionnaire (Table 3). Outcomes of the diary and the questionnaire were congruent in only 63% of the subjects (both negative 49%, both positive 14%). A relevant portion of patients (18%) reported to have SB, but did not show improvement on the diary. A comparable 19% of subjects did show motor improvement after sleep in the diary, but did not perceive themselves as having SB.

**Table 3:** **Number of patients with sleep benefit based on diary or questionnaire**

		**SB based on diary**		**Total**
		**no-SB**	**SB**	
**SB based on questionnaire**	**no-SB**	115 (72.3%)	44 (27.7%)	159
	**SB**	41 (56.2%)	32 (43.8%)	73
**Total**		156	76	232

Percentage shown: within SB based on questionnaire.

As the groups with SB according to either the diary or the questionnaire were not fully overlapping, we combined the outcomes of both instruments in an additional analysis, which indicated that both instruments may probe different aspects of SB. Patients with and without diary-determined SB, did not report differences in the characteristics and experience of their perceived SB in the questionnaire.

Moreover, unusual, ‘non-characteristic’ SB descriptions (e.g. “I feel less tired” or “I have more energy”) could be provided by patients both with and without SB according to the diary. Conversely, some patients with very clear, characteristic and convincing descriptions of SB in the questionnaire, did not show any improvement in subjective motor function related to sleep in the prospective diary.

Motor function changes after a daytime nap were larger in patients subjectively reporting SB in the questionnaire, compared to those not reporting SB (mean improvement 0.53 ± 1.54 vs. 0.06 ± 0.78 respectively, p < 0.001). A trend was found in the similar direction when looking at overnight changes in motor function (mean improvement in patients with subjective SB 0.01 ± 1.53 vs. -0.12 ± 1.06 in patients without, p = 0.052).

## Discussion

The existence of SB in PD has been reported repeatedly in the literature [1,4-6,8,10-13]. Here, we examined subjective SB in a structured, prospective design using a 7-day diary. Additionally, we compared the diary responses to a more traditional way of assessing SB using a questionnaire. As such, the present study represents the first prospective assessment of sleep benefit over a longer period of time. Using either instrument, we found a prevalence of subjective SB of just over 30% and the SB group had longer medication use and a tendency towards shorter night sleep. Remarkably, the groups with SB according to the diary and according to the self-report questionnaire were overlapping only partially. Many patients showed incongruent results on the diary and the questionnaire; they either showed improvement in the diary, but did not report to experience this improvement in the questionnaire, or they reported to experience an improvement after sleeping in the questionnaire that was not present in the diary. Nevertheless, the mean change in motor function after sleep tended to be higher in patients reporting subjective SB.

We applied a new approach in studying SB, using a diary in which structured questions about motor functioning reflected the subjective PD motor symptom severity at that moment. The diary enabled us to prospectively study patients for more than one night. Furthermore, patients did not have to indicate their general morning function, but assessed their functioning at a specific moment on specific motor domains. This was the first study that addressed over-night functioning change over a longer period of time. We chose to do this, to see whether there was any day-to-day variation in sleep-related symptom severity changes in PD. In previous research, SB has been treated as an “all or nothing” phenomenon, classifying patients as having either SB or not at all. Here we show that this is not necessarily the case. From both the diary and the questionnaire it became clear that in the majority of patients, SB was not experienced after every period of sleep. Although 29% of SB patients (according to the questionnaire) claimed that their SB was ‘always’ present, none of the patients had a positive change in functioning in all of the diary nights.

Our questionnaire provided a carefully formulated definition of SB to make it as clear as possible for the patients what we were looking for. However, the written description of their experiences indicated that some patients may still have misinterpreted the given definition. Although it was stated that SB is a specific reduction in PD symptoms, some patients confused it with more general and a-specific refreshing effects of sleep, which are not necessarily related to PD. We previously established this problem and attributed this to the ill-defined description of SB in earlier studies [9]. We feel that our strict definition increased the specificity to detect SB, which also fits with the SB prevalence of 31% in our cohort, which is slightly lower than in previous reports.

In our explanation of SB in the questionnaire, it was stated that the decrease in PD symptoms should be as good as feeling “on” (or better). However, 30% of the patients who reported SB in the questionnaire, subsequently stated that a symptom decrease was present after sleep, but not as large as when under the effect of medication. This may have caused an overestimation of SB prevalence. On the other hand, among the patients that answered negatively to the SB question, there were probably also patients who do experience some improvement after sleep, although not as large as on medication. Our data certainly indicate that there is large variation in the degree of symptom change by SB. Here -as well as in previous studies- some of the patients may have (partly) misinterpreted the SB definition, incorrectly stating to experience SB. Nevertheless, the characteristic written descriptions given by some patients, indicate that there may indeed be a group of PD patients that genuinely benefits from sleep.

Even patients with a highly characteristic and convincing description of perceived SB, did not always show sleep related symptoms changes in the diary. Therefore, the incongruence between the results from the diary and the questionnaire may further imply that these instruments assess different aspects of SB. When discussing SB, it seems necessary to make a distinction between 1) a subjective general feeling of improvement after sleep and 2) a specific improvement in actual motor functioning. Both aspects may contribute to the experiences which patients perceive as SB. The latter aspect was mostly assessed by the symptom diary, as we specifically targeted this instrument towards changes in motor function. The definition of SB in the questionnaire could be interpreted in a more general way, including non-motor symptoms of PD and/or a-specific refreshing effects of sleep. Both aspects could for sure be clinically relevant, but the underlying mechanisms are possibly different. Therefore, this distinction should be an important point of focus in future research.

We have assessed possible determinants of SB in our cohort. In both the diary- and questionnaire-determined SB we found that patients with SB had longer disease duration and longer medication use. Using the questionnaire we also found that patients with SB were younger, had a lower age of onset and used higher doses of dopaminergics. Similar results have previously been found for disease duration [5,10,11], medication use [6,11], age of onset [10,11] and LED [10,11]. Only one other study has reported a difference in age between SB and non-SB patients, but found SB patients to be older [5].

In the diary data we found a negative correlation with the magnitude of SB and self-reported sleep duration and quality. In addition, in the questionnaire a trend towards shorter sleep duration and lower sleep efficiency was present in the SB group. This possible association with shorter and/or worse nighttime sleep with the presence of SB is in line with a recent polysomnography study in which a shorter total sleep time and a longer sleep latency were found in patients experiencing SB [17]. Together, these data may suggest that SB is in fact not related to sleep, or even that sleep deprivation facilitates SB. This hypothesis has been put forward in the literature before [5,13]. When a causal relation between (good) sleep and improved motor function is abandoned, the thinking about the putative mechanism underlying SB may also have to change. The currently most common hypothesis states that dopamine storage in nigral neuronal terminals are replenished during sleep as the mediator of SB [9]. However, this would not fit with the association between poorer sleep quality and SB, although it should be noted that these results are all based on the evaluation of nocturnal sleep. SB is also reported after daytime naps [5-8]; and assessing nap related SB may shed further light on possible underlying mechanisms. Our study however, is unsuitable for making strong inferences on these mechanistic aspects.

This study had some limitations. In the diary we could only record planned naps, as it was essential that the patient completed the diary both before and after a sleep episode. We probably missed some unplanned and unnoticed sleep episodes [18]. However, as our classification of patients having SB relied on an minimum number of naps with a positive change, this could at worst have led to an underestimation of patients with SB based on daytime naps. Furthermore, self-reports of sleep quality are not always reliable and subjective to various factors including affective disorders [19]. However, previous studies on sleep benefit that included assessment of depressive symptoms did not find differences between patients with and without sleep benefit [8,17].

As patients dropping out of the study were older and may have been different with respect to disease duration or severity, one should be careful to generalize our findings to the whole PD population.

We used a minimum of 2 nights or naps with any symptom improvement as a cut-off for SB. Because this was the first time that SB was studied using a symptom diary, we had to choose cut-off values. For this particular study, we chose to be relatively sensitive and to classify any amount of ‘over-sleep improvement’ that occurred for more than one day as indicative of SB. Although arbitrary, this is at least a very clear way of defining SB, allowing comparisons between studies.

## Conclusions

We showed that the subjective experience of SB in PD is not always related to an actual increase in reported motor function after sleep. Defining SB using either a symptom diary or a questionnaire on subjective experience, probed different aspects of the phenomenon, resulting in only partly overlapping groups. These data suggest that SB may be a more heterogeneous phenomenon than previously thought and that subjective experiences of symptom severity is not necessarily related to actual motor function.

Given its potential clinical relevance as therapeutic intervention, there is now a crucial need for detailed prospective studies using quantitative objective measures of motor performance, in relation to objective assessment of sleep quality. Such studies should also monitor the effects of SB on non-motor symptoms of PD, including cognitive deficits and fatigue, as these may potentially improve in relation to sleep as well.

## Additional file

Additional file 1:
**Sleep benefit questionnaire.**

## References

[1] Comella CL (2007). Sleep disorders in Parkinson’s disease: an overview. Mov Disord.

[2] Askenasy JJ (2003). Sleep disturbances in Parkinsonism. J Neural Transm.

[3] Louter M, Aarden WC, Lion J, Bloem BR, Overeem S (2012). Recognition and diagnosis of sleep disorders in Parkinson’s disease. J Neurol.

[4] Marsden CD, Rinne U, Klinger M, Stamm G (1980). “On-off” phenomena in Parkinson’s disease. Parkinson’s Disease: Current Progress, Problems and Management.

[5] Merello M, Hughes A, Colosimo C, Hoffman M, Starkstein S, Leiguarda R (1997). Sleep benefit in Parkinson’s disease. Mov Disord.

[6] Tandberg E, Larsen JP, Karlsen K (1999). Excessive daytime sleepiness and sleep benefit in Parkinson’s disease: a community-based study. Mov Disord.

[7] Pal PK, Thennarasu K, Fleming J, Schulzer M, Brown T, Calne SM (2004). Nocturnal sleep disturbances and daytime dysfunction in patients with Parkinson’s disease and in their caregivers. Parkinsonism Relat Disord.

[8] van Gilst MM, Louter M, Baumann CR, Bloem BR, Overeem S (2012). Sleep benefit in Parkinson’s disease: time to revive an enigma?. J Park Dis.

[9] van Gilst MM, Bloem BR, Overeem S (2013). “Sleep benefit” in Parkinson’s disease: a systematic review. Parkinsonism Relat Disord.

[10] Bateman DE, Levett K, Marsden CD (1999). Sleep benefit in Parkinson’s disease. J Neurol Neurosurg Psychiatry.

[11] Currie LJ, Bennett JP, Harrison MB, Trugman JM, Wooten GF (1997). Clinical correlates of sleep benefit in Parkinson’s disease. Neurology.

[12] Factor SA, Weiner WJ (1998). ‘Sleep benefit’ in Parkinson’s disease. Neurology.

[13] Högl BE, Gomez-Arevalo G, Garcia S, Scipioni O, Rubio M, Blanco M (1998). A clinical, pharmacologic, and polysomnographic study of sleep benefit in Parkinson’s disease. Neurology.

[14] Marinus J, Visser M, Stiggelbout AM, Rabey JM, Bonuccelli U, Kraus PH (2002). Activity-based diary for Parkinson’s disease. Clin Neuropharmacol.

[15] Tomlinson CL, Stowe R, Patel S, Rick C, Gray R, Clarke CE (2010). Systematic review of levodopa dose equivalency reporting in Parkinson’s disease. Mov Disord.

[16] Buysse DJ, Reynolds CF, Monk TH, Berman SR, Kupfer DJ (1989). The Pittsburgh Sleep Quality Index: a new instrument for psychiatric practice and research. Psychiatry Res.

[17] Sherif E, Valko PO, Overeem S, Baumann CR (2014). Sleep benefit in Parkinson’s disease is associated with short sleep times. Parkinsonism Relat Disord.

[18] Bolitho SJ, Naismith SL, Salahuddin P, Terpening Z, Grunstein RR, Lewis SJ (2013). Objective measurement of daytime napping, cognitive dysfunction and subjective sleepiness in Parkinson’s disease. PLoS One.

[19] Aitken D, Naismith SL, Terpening Z, Lewis SJ (2014). Dysfunctional sleep beliefs in Parkinson’s disease: relationships with subjective and objective sleep. J Clin Neurosci.

