# Supplementary material for: Prospective assessment of subjective sleep benefit in Parkinson’s disease
Source: BMC Neurol. 2015 Jan 16;15:2. doi: 10.1186/s12883-014-0256-2 (PMC4300997; doi:10.1186/s12883-014-0256-2)
Supplement: Additional file 1: — Sleep benefit questionnaire. [file 12883_2014_256_MOESM1_ESM.docx]

**Sleep Benefit questionnaire**

The sleep benefit questionnaire used in our study was created in the Dutch language. Below we have provided a *non-validated* translation in English.

In addition to this questionnaire, we administered the Pittsburgh Sleep Quality Index (Buysse et al. 1989), and a few questions regarding demographic and clinical characteristics (age, sex, disease duration, medication details)

**Sleep and Parkinson’s disease**

*Some patients experience a clear decrease in Parkinson’s symptoms after a period of sleep. For example, at awakening they feel less stiff or slow or have less tremor, as if they have already taken their Parkinson medication.*

The following questions are about this clear decrease in symptoms after a period of sleep.

1. Are you familiar with this decrease in symptoms after a period of sleep?
   1. Yes, my Parkinson symptoms are clearly decreased after a period of sleep
   2. No, there is no difference in my Parkinson symptoms after a period of sleep (you can skip to end of the questionnaire)
   3. No, my Parkinson symptoms are worse after a period of sleep (you can skip to end of the questionnaire)
2. When do you experience a decrease in Parkinson symptoms after a period of sleep?
   1. Only after nighttime sleep
   2. Only after a daytime nap
   3. After both nighttime sleep and a daytime nap
3. How often do you experience a decrease in Parkinson symptoms after a period of sleep?
   1. Always
   2. Often
   3. Sometimes
   4. Occasionally
4. How large is the decrease in Parkinson symptoms after a period of sleep?
   1. I feel as good as when I have taken my medication
   2. I feel better than when I have taken my medication
   3. I feel good, but not as good as when I have taken my medication
5. How long does the decrease in Parkinson symptoms after a period of sleep last?

……… minutes

1. Is the decrease in Parkinson symptoms after a period of sleep still present when you have taken your first medication after sleep?
   1. I do not use Parkinson medication
   2. Yes
   3. No
2. Is the decrease in Parkinson symptoms after a period of sleep also present when you have slept *shorter* than you usually do?
   1. No
   2. Yes, but the decrease in symptoms is smaller
   3. Yes, the decrease in symptoms is the same
   4. Yes, the decrease in symptoms is larger
   5. I don’t know
3. Is the decrease in Parkinson symptoms after a period of sleep also present when you have slept *longer* than you normally do?
   1. No
   2. Yes, but the decrease in symptoms is smaller
   3. Yes, the decrease in symptoms is the same
   4. Yes, the decrease in symptoms is larger
   5. I don’t know
4. Do you also experience a decrease in Parkinson symptoms after a period of sleep *during* the night, for example when going to the toilet?
   1. Yes
   2. No
   3. I don’t know
5. Do you also experience a decrease in Parkinson symptoms after a period of calm wakefulness (e.g. sitting in a chair, without sleeping)?
   1. No
   2. Yes, but the decrease in symptoms is smaller than after a period of sleep
   3. Yes, the decrease in symptoms is the same as after a period of sleep
   4. Yes, the decrease in symptoms is larger than after a period of sleep
   5. I don’t know
6. Does a period of sleep influence the effect of your Parkinson medication? (multiple answers possible)
   1. I do not use Parkinson medication
   2. No
   3. Yes - after a period of sleep my medication works better
   4. Yes - after a period of sleep my medication works faster
   5. Yes - after a period of sleep my medication works not as good as normal
   6. Yes - after a period of sleep my medication works slower
7. You have answered some questions about a decrease in Parkinson symptoms after a period of sleep. Because Parkinson’s disease is different for every patient, the experiences of symptoms after a period of sleep is probably different as well. Can you describe in your own words how you feel at awakening and what the decrease in Parkinson symptoms after a period of sleep means to you?

………………………………………………………………………………………………………………………………………………………………………………………………………………………………………………………………………………………………

………………………………………………………………………………………………………………………………………………

………………………………………………………………………………………………………………………………………………

………………………………………………………………………………………………………………………………………………

………………………………………………………………………………………………………………………………………………
